# Occupational Health among Swedish Occupational Therapists: A Cross-Sectional Study

**DOI:** 10.3390/ijerph17103379

**Published:** 2020-05-12

**Authors:** Annika Lexén, Ida Kåhlin, Lena-Karin Erlandsson, Carita Håkansson

**Affiliations:** 1 Department of Health Sciences, Lund University, P.O. Box 157, SE-221 00 Lund, Sweden; 2 Faculty of Health Sciences, Linköping University, SE-581 83 Linköping, Sweden; ida.kahlin@liu.se; 3 Academy of Health and Welfare, Halmstad University, SE-301 18 Halmstad, Sweden; lena-karin.erlandsson@hh.se; 4 Division of Occupational and Environmental Medicine, Lund University, SE-221 00 Lund, Sweden; carita.hakansson@med.lu.se

**Keywords:** Lund University Checklist for Incipient Exhaustion, self-rated exhaustion disorder, occupational balance questionnaire

## Abstract

The Swedish public sector is facing great challenges in recruiting and retaining healthcare professionals, due to increasing sick leave numbers. The aim of this study was to describe Swedish occupational therapists’ occupational health in terms of risk factors in the social and organizational work environment, occupational balance, and work-related mental health problems. A web survey was emailed to all working members of the Swedish Association of Occupational Therapists (*n* = 7600) and 3658 answered the survey. The web survey included questions on sociodemographic characteristics, social and organizational environment, occupational balance, and work-related health. The occupational therapists in general rated their workload as high, which was described as leading to increased stress, difficulties doing a good job, and increased job turnover. They also reported having difficulties maintaining occupational balance. Almost a fifth reported having symptoms related to mild incipient exhaustion or a pronounced exhaustion disorder. Almost 60 percent reported having, during the last year, seriously considered seeking new employment as an occupational therapist and 35 percent had seriously intended to leave their profession. In conclusion, there is an urgent need to improve the work situation of occupational therapists. If not, increases in mental health problems, sick leave and job turnover rates may seriously jeopardize the welfare system by eroding healthcare.

## 1. Introduction

In Sweden, as in most European countries, mental health problems are increasing, especially among individuals of working age [1]. Sweden is, however, distinguished from other European countries by the fact that sickness absence rates have increased sharply over time, causing a huge burden on Swedish society [2]. In Sweden, between 2011 and 2017, the amount of sick leave due to mental health problems increased by 129 percent and now constitutes 45.2 percent of the total number of cases of sick leave [3]. Sweden’s public healthcare service, in particular, is facing great challenges in recruiting and retaining healthcare professionals, due to high sick leave numbers because of mental health problems [2,4]. These challenges are amplified by the fact that many professionals within healthcare services are close to retirement [5]. At the same time, with comparatively fewer healthcare professionals available, more healthcare needs to be provided due to demographic changes, with an increasing number of elderly people in the Swedish population. This is a major societal problem, threatening the maintenance of the Swedish welfare system [2].

According to the Organization for Economic Co-operation and Development [6], job strain is the most common cause of work-related mental health problems among Swedish employees today. There is compelling evidence that the quality of the work environment can erode mental health with sickness absence as a result [7]. Known risk factors are, for example, high demands and little control over the work situation, unclear roles, lack of leadership, imbalance between effort and reward, and lack of support from manager and colleagues [8]. The main reasons for an increase in mental health problems are changes in the work tasks because of increased demands, high expectations, and increasingly slim organizations [2,9,10]. In this study, mental health problems are defined in opposite terms to the definition of mental health issued by the European Union and the 3rd EU Health Program:

“… a state of well-being in which every individual realizes his or her own potential, can cope with the normal stresses of life, can work productively and fruitfully, and is able to make a contribution to her or his community”

[7] (p. 4).

In recent decades, work contexts have also changed to increasingly include more computer-based tasks and an expected ability to simultaneously handle several challenges [11]. Another contributing factor to the increase in mental health problems regards challenges in combining work with responsibilities related to home and having a family [8]. Despite increased gender equality in Swedish society [12], work-life balance challenges are still more commonly experienced among women [1,13]. Lack of occupational balance, i.e., the experience of not having the right amount and variety of activities in daily life (work, home chores, leisure, rest and sleep), has been shown to be a contributing factor for developing mental health problems [14,15].

Working in healthcare services has also been shown to be connected to an increased risk of sick leave [1,6,10]. Today, women outnumber men in the healthcare workforce [16]. For example, in the year 2017, women accounted for 88 percent of nurses, the largest group of health professionals in Sweden, and 94 percent of occupational therapists [17]. Working in healthcare services usually includes work with high psychological and emotional stress in which the professional has many relationships with other people [6,18]. Increased job strain can result in exhaustion and disengagement from work, which can negatively affect the quality of the care provided [19]. Examples of professions working in the Swedish healthcare service are social workers, psychologists, nurses, and occupational therapists [6,20]. In a study by Peterson et al. [19], the highest proportion of exhaustion among Swedish healthcare workers was found among paramedics (psychologists, social workers, occupational therapists and physiotherapists) (37.1%). Thus, there is a need to further investigate and describe the occupational health situation for occupational therapists and other groups of paramedics. The concept of occupational health deals with all aspects of health and safety in the workplace [21]. However, in this study, we focus on the risk factors in the social and organizational work environment, occupational balance, and the person’s reported work-related mental health problems, in terms of stress and exhaustion.

Work-related mental health problems have been reported among occupational therapists in several countries [22]. In a mixed method study by Gupta et al. [22], burnout or exhaustion disorder symptoms were explored among Canadian occupational therapists, with 34.8 percent of 63 participating occupational therapists experiencing high levels of exhaustion and 24.6 percent low professional efficacy, which is considered to increase the risk of developing an exhaustion disorder. In a study among 951 occupational therapists in Australia [23], factors associated with mental health problems among occupational therapists were found on both organizational and sociodemographic levels, and included having children at home, dissatisfaction with income, having less than 10 years of work experience, difficulties letting go of work-related thoughts after working hours, experience of having too much to do, difficulty in saying no, and fewer opportunities for a good laugh during working hours. Furthermore, a Swedish study among 472 occupational therapists showed that lack of work-related resources and time were the largest stress factors [24]. To the authors’ knowledge, few Swedish studies have focused on describing occupational therapists’ occupational health in terms of risk factors in the social and organizational work environment, occupational balance and work-related mental health problems (stress and exhaustion). This type of study is important to be able to rule out the extent of the problem/situation and ascertain what can be done to prevent occupational health problems in this group of healthcare professionals. Further investigation of these areas seems extraordinarily important against the background that earlier research concerning the health of occupational therapists [25], as well as among healthcare professionals in general [26,27], shows that mental health problems, such as feeling stressed or emotionally exhausted, can lead to an increase in job changes and job turnover rates. This type of research is lacking in Sweden, although mental health problems such as stress and exhaustion are an extensive public health problem that affects many individuals and society in general [8], and the recruitment and retention of professionals in healthcare specifically [2,4].

The overall aim of this study was to describe Swedish occupational therapists’ occupational health in terms of risk factors in the social and organizational work environment, occupational balance, and work-related mental health problems. The specific research questions are as follows. How do Swedish occupational therapists: (1) perceive risk factors in their social and organizational work environment?; (2) rate their occupational balance?; (3) rate their work-related mental health in terms of stress and exhaustion?

## 2. Materials and Methods

### 2.1. Overall Design

This study used a cross-sectional design. A web survey was forwarded to all currently working members of the Swedish Association of Occupational Therapists in February 2018.

### 2.2. Participants

A goal-directed sample [28] was used. The criterion for selection of participants was being a working member of the Swedish Association of Occupational Therapists. The exclusion criterion was being on sick-leave, on parental leave, unemployed, or retired. The Swedish Association of Occupational Therapists is both a trade union and a professional organization with responsibility for promoting the occupational therapy profession. The great majority (about 75 percent) of Swedish occupational therapists are members of this organization. An e-mail with information on the study and a link to the web survey was sent to all members of the Swedish Association of Occupational Therapists who meet the study criterion (*n* = 7600) and 3658 gave their informed written consent to participate in the study. This gives a response rate of 48 percent.

### 2.3. Data Collection

Data was collected using a web survey distributed via Research Electronic Data capture (REDcap), which is a secure web application for building and distributing web surveys [29]. A link to the web survey was sent to the participants’ e-mail addresses from the member list, together with an information letter describing the study aim and procedures. All procedures were in accordance with the ethical standards of the responsible committee on human experimentation (institutional and national) and with the Helsinki Declaration of 1975, as revised in 2000. The Regional Ethical Board in Lund, Sweden (2017/975) approved the study.

As a first question in the web survey, the participant was asked for informed consent. The survey was accessible for three weeks and reminders to fill in the survey were sent out on two occasions. The web survey included questions on social and organizational work environment, occupational balance, and mental health problems (stress and exhaustion). The survey also contained questions on sociodemographic characteristics including age, gender, occupational therapy education degree, work experience in years, employment degree, sector, and work position.

#### 2.3.1. Social and Organizational Work Environment, and Job Turnover

General questions about the participant’s perceptions of their social and organizational work environment and job turnover were also asked. These questions were developed to capture work-related issues of specific importance for occupational therapists, in collaboration with lawyers at the office of the Swedish Association of Occupational Therapists, who have experience of consulting and supporting members in distress-related work issues. Questions used in this part of the survey are described in Table 1.

Participants who rated their workload as high or too high were asked to consider whether they had noted the following as a consequence of the high workload: (1) increased stress, (2) difficulties doing a good job, (3) increased job turnover, (4) vacancies, (5) increased amount of sick leave, (6) difficulties getting experienced personnel, (7) increase in threats and violence, or (8) no, none of the above. Several response options were possible. Participants who filled in the last answer option “no, none of the above” were asked to write down other reasons for their rated high workload.

The web survey also included the Questionnaire for Psychological and Social factors at work (QPS)-Mismatch self-assessment [30] to assess the occupational therapists’ experiences of risk factors in their social and organizational work environment. QPS-Mismatch includes questions from the QPS Nordic general questionnaire [31] for psychological and social factors at work and concerns the match between the worker and the workplace, in keeping with Maslach and Leiter’s model of burnout/exhaustion disorder. It includes 38 questions and builds on the theory that exhaustion disorder is a result of lack of match between the worker and his or her resources and expectations, and the work characteristics according to the following dimensions: (1) workload, (2) control, (3) community in the workplace, (4) reward, (5) justice, and (6) values. The poorer the fit between the worker and the work characteristics, the bigger the risk of experiencing exhaustion disorder. Each item is rated on a 5-point scale ranging from (1) “very seldom or never” to (5) “very often or always”. Cronbach’s alpha for this study was 0.94.

#### 2.3.2. Occupational Balance

Occupational balance was assessed using the Occupational Balance Questionnaire (OBQ11) [32,33]. OBQ11 assesses the individual’s self-rated degree of occupational balance, i.e., the experience of having the right amount and variation in activities in daily living [34]. The instrument consists of 11 statements measuring different aspects of occupational balance. Every statement is rated on a scale ranging from 0 to 3, with a sum score of 33. The higher the points, the higher the experience of occupational balance. OBQ11 has been shown to have good reliability with a Pearson separation index of 0.92, and sufficient model fit and measurement invariance (construct validity) across age and gender [33]. Cronbach’s alpha for this study was 0.94.

#### 2.3.3. Mental Health Problems (Stress and Exhaustion)

The Lund University Checklist for Incipient Exhaustion (LUCIE) [35] was used for early identification of work-related stress symptoms, prodromal stages of work-related exhaustion, and exhaustion disorder [36]. The LUCIE checklist [35] consists of 28 items describing behaviors and feelings connected to prodromal stages of exhaustion disorder. The participants were asked “For the past month, to what extent have you felt or observed the following?” The items cover the following six domains: (1) sleep and recovery, (2) separation between work and spare time, (3) sense of community and support in the workplace, (4) managing work tasks and personal capabilities, (5) private life and spare time activities, and (6) health complaints. Each item is rated on a 4-point scale, ranging from (1) “not at all” to (4) “very much”. According to LUCIE, the data is then analyzed based on two algorithms of two scales: The Stress Warning Scale (SWS), which indicates milder signs of incipient exhaustion, and the Exhaustion Warning Scale (EWS), indicating severe signs of exhaustion. The scales’ scores range from 0 to 100. An SWS ≤ 17.00 indicates no or negligible lasting stress symptoms, SWS 17.01 to 38.5 possible slight lasting stress symptoms, and SWS ≥ 38.51 mild to moderate stress symptoms. An EWS ≤ 21.50 indicates that signs of exhaustion are mostly absent or mild and EWS > 21.50 suggests severe symptoms and indicates exhaustion disorder. When combining the scores from the two scales, they together provide a 4-step severity ladder of stress symptomatology: (1) no or negligible lasting stress symptoms, (2) possible lasting stress symptoms, (3) mild to moderate lasting stress symptoms, and (4) lasting stress symptoms of a severity indicating possible exhaustion disorder. LUCIE has been found to be a clinically relevant tool for the clinical screening of incipient work-related exhaustion [35,36]. LUCIE has, in previous studies, been compared to other relevant measures such as the Karolinska Exhaustion Disorder Scale (KEDS) and the Self-rated Exhaustion Disorder (s-ED) scale [35]. The extent to which LUCIE can detect the onset of sustained incipient exhaustion has also been evaluated with good results [36]. Cronbach’s alpha for this study was 0.78.

The Self-rated Exhaustion Disorder (s-ED) scale [37] was used to detect symptoms of exhaustion disorder. The instrument is based on the clinical diagnostic criteria for exhaustion disorder, as proposed by the Swedish National Board of Health and Welfare. In this instrument, the person makes a self-rating of the diagnostic criteria for exhaustion disorder. It consists of four questions, one of which comprises 6 sub-questions: (1) concentration difficulties or impaired memory; (2) markedly reduced capacity to deal with demands or to work under time pressure; (3) emotional instability or irritability; (4) sleep disturbances; (5) marked physical weakness or fatigability, and; (6) physical symptoms, such as muscular pain and chest pain. In a previous research study, s-ED proved to have sufficient construct validity, i.e., to correspond well to established scales measuring mental health problems [37]. Cronbach’s alpha for this study was 0.73.

### 2.4. Statistical Methods

All analyses were performed using SPSS Statistics 25.0 (IBM Corps., Armonk, NY, USA). Cronbach’s alpha was calculated to assess the used instruments’ reliability, or internal consistency. Descriptive statistics, including frequencies (*n*), percentages (%), medians (mdn), average scores (M), and standard deviations (SD) were used to analyze the results. In addition, for the QPS-Mismatch instrument, z-values were used to make comparisons possible with the QPS Nordic reference group average score [31].

## 3. Results

### 3.1. Sociodemographic Characteristics

Most participants were women (94%), middle-aged (m = 45 ± 11) working more than 75 percent of full-time (40 h) (89%). Most of them worked within the Swedish healthcare service (85%). For more information on sociodemographic factors, see Table 2.

### 3.2. Social and Organizational Work Environment, and Job Turnover

#### 3.2.1. Possibilities for Professional Development and Support from Occupational Therapy Colleagues

About half of the participants (49%, *n* = 1805) were very or quite dissatisfied with the possibilities for professional development. On a scale ranging from very dissatisfied (1) to satisfied (5), the participants’ median score was 3.00 and average score 3.38 (SD = 0.94). Seventy-four percent (*n* = 2699) of the participants were satisfied or very satisfied with the support from their occupational therapy colleagues (mdn = 4.00, M = 4.13 ± 0.91 out of 5).

#### 3.2.2. Seeking New Employment or Intention to Leave the Profession

Fifty-eight percent (*n* = 2133) reported that, during the last year, they had seriously considered seeking new employment as an occupational therapist and 35 percent (*n* = 1279) had seriously considered leaving their profession. Respondents within the healthcare service accounted for the largest proportion of respondents, 86 percent, who had seriously considered seeking another job or intended to leave the profession.

#### 3.2.3. Workload

Sixty-seven percent (*n* = 2282) of the participants rated their workload as high or too high. On a scale ranging from (1) “Even if the workload increased there is room to complete my work tasks” to (5) “We have too high a workload and not enough time to complete our work tasks”, the participants’ median score was 4.00, with a mean score of 3.75 (SD = 0.81). This was described as leading to consequences in terms of, for example, increased stress, difficulties doing a good job, and increased job turnover (Table 3).

Seventy-nine percent (*n* = 2691) stated that they did not have time to finish their work tasks by the end of the working day (quite often, often and all the time). On a scale ranging from (1) “All the time” to (5) “Never”, the participants’ median score was 3.00, with a mean score of 3.32 (SD = 0.93).

#### 3.2.4. Work Environment Risk Factors

The participants’ ratings on QPS-Mismatch are on a group level within a standard deviation in relation to the reference group in QPS Nordic average scores, i.e., above average on a normal distribution curve. All sub-scales were above average in relation to the reference group, except for “reward” and “justice”, where the respondents scored below average, i.e., the respondents generally felt that they did not receive enough reward for a job well done and that their values did not always match the values of the workplace. Participants who scored on s-ED as having a mild or a pronounced exhaustion disorder scored between 0 and −1 standard deviations (below average) in relation to the reference group’s average scores on all QPS-Mismatch sub-scales, i.e., workload, control, community in the workplace, reward, justice, and values. For more information, see Table 4.

### 3.3. Occupational Balance

The participants’ median score was 13 (M = 14.1 ± 7) of a total score of 33 on OBQ11. The median score for all items was 1.00 and average scores ranging from 1.19 to 1.42 on a scale ranging from 0 to 3.

### 3.4. Mental Health Problems (Stress and Exhaustion)

According to the ratings on the LUCIE instrument 40 percent (*n* = 1305) of the participants reported no or negligible lasting stress symptoms (SWS > 17.00). Eighteen percent (*n* = 571) reported mild to moderate stress symptoms (SWS > 38.51) indicating milder signs of incipient exhaustion and 7 percent (*n* = 236) lasting stress symptoms that might indicate a severe exhaustion disorder (EWS > 21.50).

When combining the scores from the SWS and EWS scales in LUCIE to analyze the severity of stress symptomatology, 60 percent (*n* = 1953) of the participants reported no or negligible symptoms, 22 percent (*n* = 712) possible lasting stress symptoms, 11 percent (*n* = 357) mild to moderate lasting stress symptoms, and 7 percent (*n* = 214) lasting stress symptoms of a severity indicating a possible exhaustion disorder.

Twenty-one percent (*n* = 750) of the participants met the Swedish National Board of Health and Welfare diagnostic criterion for mild (10.4%, *n* = 379) or pronounced (10.2%, *n* = 371) exhaustion disorder, according to the s-ED instrument.

## 4. Discussion

This study focused on occupational therapists and almost 50 percent of the working members of the Swedish Association of Occupational Therapists participated. A clear majority were women, with a mean age of 45 years, mainly working within the Swedish healthcare service. The occupational therapists in the present study reported having a high workload, difficulty maintaining occupational balance, and signs of work-related mental health problems in terms of prodromal stages of exhaustion and exhaustion disorder.

### 4.1. Social and Organizational Work Environment, and Mental Health

The rated high workload among occupational therapists in the present study indicates an obvious risk factor for mental health problems, sick leave, and job turnover in the group, in keeping with previous studies [22,25]. The experience of having too much to do has been found to be associated with exhaustion disorder among occupational therapists [22]. This in turn may lead to an increase in job changes and job turnover rates and a deteriorating healthcare service, in keeping with a previous study [25]. In the present study a great proportion of the occupational therapists had seriously considered changing job, or had intended to leave their profession. Forty percent (40%) reported having stress symptoms and almost a fifth (20%) as having symptoms related to mild incipient exhaustion or a pronounced exhaustion disorder. These results are in line with previous research [15,19,22]. In the recently published study by Håkansson and Ahlborg [15] of 2223 employees in public organizations in Sweden, 25 percent of the women and 17 percent of the men had an exhaustion disorder. In the study by Gupta and colleagues [22], a great proportion of the occupational therapists included (34.8%) experienced a high level of stress and exhaustion, indicating a risk of developing a pronounced exhaustion disorder. Furthermore, Peterson et al. [19] found that the highest proportion of exhaustion among Swedish healthcare workers was among paramedics (psychologists, social workers, occupational therapists and physiotherapists) (37.1%). Accordingly, these results are alarming and should be considered by employers and policy makers who are expected to guarantee the quality of the professionals’ work being done, as well as provide a good social and organizational work environment and prevent risks of ill health due to social and organizational conditions, as described by the Swedish Work Environment Authority [9]. In a report from the European Agency for Safety and Health at Work [38], these risks were raised as a challenge for the third parties in ensuring the required level of healthcare services. Accordingly, there is a need to address risk factors in the social and organizational work environment and mental health among occupational therapists and other healthcare professionals to promote and protect well-being and mental health in these groups.

The fact that during the last year, a great proportion of the occupational therapists in the present study had seriously considered changing jobs, or had intended to leave their profession, needs to be taken seriously. The reasons for job turnover among occupational therapists have been addressed in a previous study by Scanlan and Still [25]. They found that exhaustion disorder was associated with lower job satisfaction and higher turnover rates among 103 occupational therapists working within mental healthcare. There might be an imminent risk that the healthcare service in Sweden will lose experienced occupational therapists and this, in turn, will affect the healthcare given. There is a need to further explore occupational therapists’ reasons for considering changing job and/or leaving their profession. There is also a need to explore how occupational therapists that are new to their profession feel about their mental health, job strain, and intention to leave the profession.

Seventy-four percent of the participants in the present study were satisfied with the support from their occupational therapy colleagues. According to the job demand-control-support model, balance between demands, decision latitude and social support is vital to experiencing mental health at work [39,40]. However, although this is a positive result, high demands and low decision latitude in the group might still increase the risk of developing mental health problems.

According to QPS-Mismatch, the respondents generally stated that they did not receive enough reward for a job well done and that their values did not always match the values of the workplace. In previous research, this has been shown to create conflicts and increase the psychosocial workload, which in turn can lead to mental health problems [8,10]. According to Maslach and Leiter’s [41] theory, stress and exhaustion are the result of a lack of fit between an individual and their resources, expectations at work, and the actual nature of work in the areas of workload, control, community in the workplace, reward, justice, and values. The participants in the present study who rated on s-UMS as having symptoms of mild or pronounced exhaustion generally rated below average in relation to the QPS Nordic reference group’s average score on all QPS-Mismatch sub-scales, i.e., workload, control, community in the workplace, reward, justice, and values. Workplaces where occupational therapists are employed generally need to work preventively to a higher degree to prevent work-related mental health problems. A viable strategy is to develop and implement a mental health and well-being strategy and a policy on workplace prevention of mental health problems [42]. According to the workplace prevention of mental health problems guidelines for organizations [42], the goal should be to create a positive working environment that provides the conditions for the employee to balance demands and control in the work situation, by clarifying roles and responsibilities and ensuring a manageable workload. It is also important to reward the efforts of occupational therapists and other employees through, for example, a salary that the employee perceives as fair (justice) and clearly related to the employer’s competence and educational level. The opportunity for professional development is also important. About half of the participants in the present study were dissatisfied with the possibilities for professional development. In line with Toomingas et al. [43], a work environment that promotes mental health and well-being is of great importance, not least because we spend a large part of our waking time at work. However, the area is described as complex, as the origin of mental health problems can be found in both work and private life [44].

### 4.2. Occupational Balance

The great majority of the respondents scored between 1.19 and 1.42 on a scale ranging from 0 to 3 on the individual items on the OBQ11 questionnaire, indicating an oblique distribution of the scored statements, where most participants stated that they did not fully agree or just partially agreed with the statements in OBQ11. The participants’ median score for the sum score was 13 (possible total score 0–33). This is in keeping with a previous study using OBQ11 among university teachers in Sweden, where the median occupational balance sum score was 11 [45], and a study of women in the general Swedish population (mean age of 41 years), where the median OBQ11 sum score was 12 [46]. As in the present study, the participants in these two studies were mainly women and their median occupational balance sum score indicates difficulties maintaining occupational balance, i.e., the experience of not having the right amount and variety of activities in daily life (work, home chores, leisure, rest and sleep). Difficulties maintaining occupational balance [15,47,48] and work-life imbalance [8,10] are known risk factors for developing mental health problems, especially among women. The importance of occupational balance for health among occupational therapists is also highlighted by Wagman et al. [47] and described by AFA Insurance [4] as a possible cause of increased sick leave due to mental health problems, mainly in professions that include work in which the professional has many relationships with other people, such as within the healthcare sector. A study by Borgh et al. [48] investigating the association between organizational factors such as workplace culture and climate, and the experience of having occupational balance, showed that parents with young children who felt that colleagues and employers had positive attitudes towards parenthood and parental leave experienced occupational balance to a greater extent than parents who were met by negative or neutral attitudes from colleagues and employers. There was also a strong association between clear routines for handling job duties if absent, and occupational balance among the study participants. Based on the present study, it might be concluded that it is important that workplaces create conditions for and support the individual employee’s occupational balance in relation to the employee’s life outside work and from a life phase perspective. In this way, the organization’s capacity to promote mental health may increase. There is also a need to further explore associations between the combination of occupational therapists’ working conditions, occupational balance, and health.

### 4.3. Methodological Considerations

This was a cross-sectional study indicating severe work-related issues, risk of mental health problems, and sick leave among occupational therapists. There appears to be an urgent need for a follow-up to explore any changes over time or development of the work situation among occupational therapists in Sweden. A limitation of this study is the response rate of 48 percent. A risk when sending out the same email to many people at the same time is the risk of email hosts blocking or blacklisting as a defense against spammers [49]. This might have resulted in not all the 7600 occupational therapists receiving the email containing the link to the web survey. However, the risk of response bias in this study can be considered as low and the number of occupational therapists answering the study was enough to give a nuanced result. Furthermore, a descriptive study limits the possibility to draw conclusions based on the results, because of the lack of statistical testing. Additionally, as made in the present study, asking the participating occupational therapists to rate consequences of their high workload is not a reliable measure. There is therefore a need for further research exploring the correlations and associations between risk factors in the social and organizational work environment, occupational balance, and mental health problems (stress and exhaustion) among occupational therapists in Sweden. However, according to Kazdin [28], the advantage of descriptive research is that it gives an overview of a phenomenon and provides the possibility to observe a research phenomenon in its natural and unchanged environment, which the present study intended to do. Another limitation is that QPS-Mismatch has not been properly psychometrically evaluated. The reliability of the scale has been found to be good with a Cronbach’s alpha value of 0.72–0.90 for individual dimensions [50]. However, it seemed necessary to include this instrument, as it reflects the nature of the risk factors to which the individual is exposed in the work environment in contrast to LUCIE, which reflects how the individual answers/reacts to different factors in the work environment. Furthermore, QPS-Mismatch was developed based on the QPS Nordic and the extensive amount of research on social and organizational factors at work that affect the mental health of an employee [30,31]. QPS Nordic has been found to have sufficient psychometric properties for assessing health-related factors at work among employees in Sweden [51]. A limitation is, however, that the QPS Nordic reference group data used to analyse the data in QPS-Mismatch contains mixed occupational groups, which may also have affected the results. Accordingly, the lack of psychometric evidence for QPS-Mismatch and the mixed occupational groups in the reference group data need to be considered when reading the results.

## 5. Conclusions

The results of this study showed that the Swedish occupational therapists participating in this study in general rated their workload as high and most of them had difficulties finishing their work tasks by the end of a working day and maintaining occupational balance. The high workload was described as leading to consequences in terms of increased stress, difficulties doing a good job, and increased job turnover. Almost a fifth of the participating occupational therapists reported having mental health problems in terms of symptoms of mild incipient exhaustion or pronounced exhaustion disorder. Almost 60 percent had, during the last year, seriously considered seeking new employment as an occupational therapist and 35 percent had seriously considered leaving their profession. The healthcare service in Sweden is a highly knowledge-driven sector when it comes to promoting health and well-being for its users. However, to maintain the welfare system as demographic changes occur, organizations within this sector need to be informed by research also related to risk factors in the social and organizational work environment of employees. It can be used to inform organizations when developing healthcare services not only to promote the health of their service users, but also of their employees. If sick leave due to mental health problems among occupational therapists and other professionals in Swedish welfare services continues to rise, it may seriously jeopardize the welfare system.

## Figures and Tables

**Table 1:** General questions related to the social and organizational work environment, and job turnover included in the web survey.

Questions	Rating Scale
How satisfied are you with the possibilities for professional development?	Five-point scale ranging from (1) dissatisfied to (5) satisfied
How satisfied are you with the support from your occupational therapy colleagues?	Five-point scale ranging from (1) dissatisfied to (5) satisfied
In general, how do you experience the workload at your workplace?	Five-point scale ranging from (1) even if the workload increased there is room to complete my work tasks to (5) we have too high a workload and not enough time to complete our work tasks
Does it happen that you do not have time to finish what you started during the working day?	Five-point scale ranging from (1) all the time to (5) never
In the past year, have you ever seriously considered seeking a new position?	Two-point scale: (1) Yes, (2) No
During the past year, have you seriously considered leaving your profession and doing something completely outside of your occupational therapy profession?	Two-point scale: (1) Yes, (2) No

**Table 2:** Description of sociodemographic factors for occupational therapists participating in the web survey (*n* = 3658).

Sociodemographic Factors	*n*	%
Sex (*n* = 3529)		
Male	186	5
Female	3332	94
Other	11	1
Age, M (±SD) (*n* = 3504)	45 (±11)	
Occupational therapy education degree (*n* = 3531)		
Professional/bachelor’s degree	3193	90
Master’s degree	261	8
Doctoral degree	73	2
Work experience (number of years), M (±SD) (*n* = 3492)	18 (±11)	
Employment degree (*n* = 3531)		
<49%	39	1
50–75%	321	10
>75%	2991	89
Sector (*n* = 3517)		
Municipality and region (healthcare services)	3008	85
Private	306	9
State (i.e., university, public employment service)	200	6
Position (*n* = 3414)		
Occupational therapist	3007	88
Manager	175	5
Researcher and/or lecturer at university	62	2

**Table 3:** Reported consequences of a heavy workload among occupational therapists in Sweden (*n* = 3658).

Consequences of a Heavy Workload ^1^	*n*	%
Increased stress	1924	47
Difficulties doing a good job	1616	44
Increased job turnover	1011	28
Vacancies	966	26
Increased amount of sick leave	825	23
Difficulties getting experienced personnel	794	22
Increase in threats and violence	47	1

^1^ Several response options were possible.

**Table 4:** Deviations in psychosocial workload among Swedish occupational therapists in relation to the QPS Nordic ^1^ reference group’s average score in the group as a whole and on sub-group levels among those who reported having symptoms of mild or pronounced exhaustion (*n* = 3658).

QPS-Mismatch ^1^—Psychosocial Workload	Whole Group (*n* = 3658)			Mild Incipient Exhaustion ^2^ *n* = 379		Pronounced Exhaustion ^2^ *n* = 371	
Dimensions	*n*	M (SD)	z-Value ^3^	*n*	z-Value ^3^	*n*	z-Value ^3^
(1) Workload	3252	3.5 (0.8)	0.29	370	−0.06	363	−0.23
(2) Control	3250	3.1 (0.4)	0.02	369	−0.27	363	−0.40
(3) Reward	3228	2.2 (1.0)	−0.14	371	−0.29	363	−0.48
(4) Community in the workplace	3254	3.1 (0.6)	0.12	368	−0.10	362	−0.29
(5) Justice	3241	2.9 (0.9)	0.07	371	−0.14	361	−0.31
(6) Value	3252	2.6 (0.6)	−12	371	−0.37	363	−0.52

^1^ QPS-Mismatch [30]; ^2^ Self-rated Exhaustion Disorder (s-ED) [37]; ^3^ QPS Nordic [30,31]; QPS—Questionnaire for Psychological and Social factors at work; M—average scores; SD—standard deviation.
